# Genome assembly at chromosome scale with telomere ends for Pearlspot, *Etroplus suratensis*

**DOI:** 10.1038/s41597-024-04096-0

**Published:** 2024-11-13

**Authors:** Vinaya Kumar Katneni, Karthic Krishnan, Sudheesh K Prabhudas, Roja Jayaraman, Nida Quraishi, Kumaraguru Vasagam, Ashok Kumar Jangam, Jesudhas Raymond Jani Angel, Nimisha Kaikkolante, Kumaravel Jayaraman, S Shekhar Mudagandur

**Affiliations:** 1 Centre for Bioinformatics, Nutrition Genetics and Biotechnology Division, ICAR - Central Institute of Brackishwater Aquaculture, No 75, Santhome High Road, MRC Nagar, Chennai, 600 028 Tamil Nadu India; 2 Nutrition Genetics and Biotechnology Division, ICAR - Central Institute of Brackishwater Aquaculture, No 75, Santhome High Road, MRC Nagar, Chennai, 600 028 Tamil Nadu India; 3 Crustacean Culture Division, ICAR-Central Institute of Brackishwater Aquaculture, No 75, Santhome High Road, MRC Nagar, Chennai, 600028 Tamil Nadu India; 4 Aquatic Animal Health and Environment Division, ICAR-Central Institute of Brackishwater Aquaculture, No 75, Santhome High Road, MRC Nagar, Chennai, 600028 Tamil Nadu India

**Keywords:** Genome informatics, Genomics

## Abstract

The pearlspot, *Etroplus suratensis* is a climate resilient cichlid fish that exhibits unusual adaptation to salinity. The fish is able to complete full life cycle in diverse salinity habitats ranging from fresh water to marine environments. High-quality primary and phased genome assemblies were generated for pearlspot fish using PacBio HiFi and Arima HiC sequencing technologies, for the first time. The primary assembly is highly contiguous with contig N50 length of 36 Mb. The final assembly is of 1.247 Gb with N50 length of 51.57 Mb and 98% of the genome length anchored to 24 chromosomes. The genome was assessed to be 99.9% complete based on BUSCO evaluation and was predicted to contain 52.96% repeat elements. We have predicted 27,192 protein encoding genes, of which 21,580 were functionally annotated. The genome offers an invaluable resource to understand adaptation of pearlspot fish to diverse salinity habitats.

## Background and Summary

Cichlid fishes characterized by rapid adaptive radiation and sympatric speciation^1^ serve as excellent model species to understand divergent evolution. Existence of vast number of closely related cichlid species within the confines of a single geographical environment^2^ displaying wide phenotypic variation makes them an ideal model system to understand the genetic basis of vertebrate speciation^3^. While the genomic resources of African cichlid fishes of the subfamily Pseudocrenilabrinae are being extensively used to understand vertebrate speciation, the resources for Asian cichlid fish of subfamily Etroplinae are scanty.

**Fig. 1:**
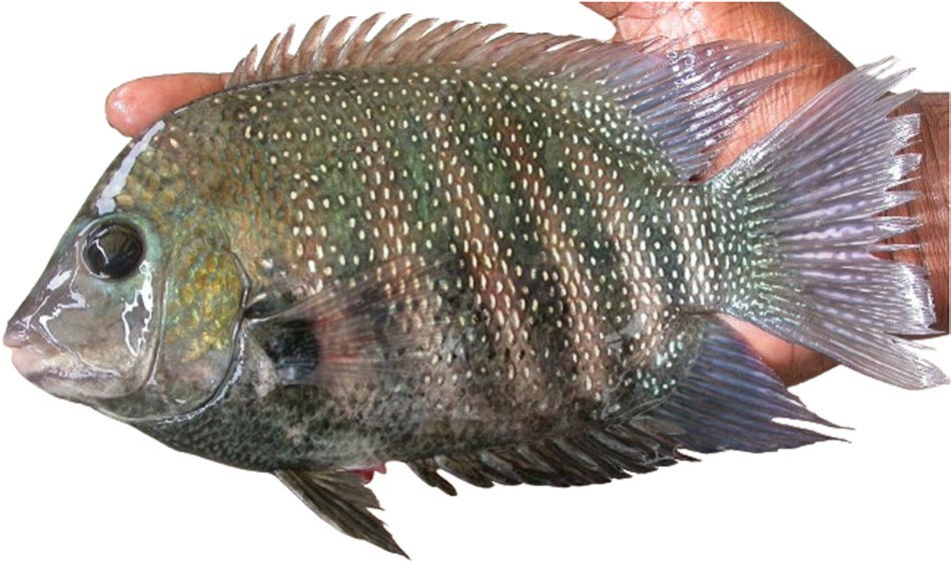
A specimen of *Etroplus suratensis*.

The *Etroplus suratensis* (Bloch, 1790) commonly known as pearlspot or green chromide is an edible fish of the subfamily Etroplinae. This substrate spawning cichlid fish (Fig. 1) is characterized by elaborate courtship and multiple parental care^4^. Though brackishwater is principal habitat for this herbivorous fish, it displays great adaptations to salinity by surviving and breeding in freshwater habitats. Only two species of the subfamily Etroplinae, *Etroplus canarensis* and *Paratilapia polleni* have draft genome assemblies available in public repository^5^. Both of these assemblies were generated with short DNA reads and have thousands of scaffolds with N50 lengths around 20 Kb. Therefore, at present, we do not have a chromosome-scale reference genome for the subfamily *Etroplinae*.

**Fig. 2:**
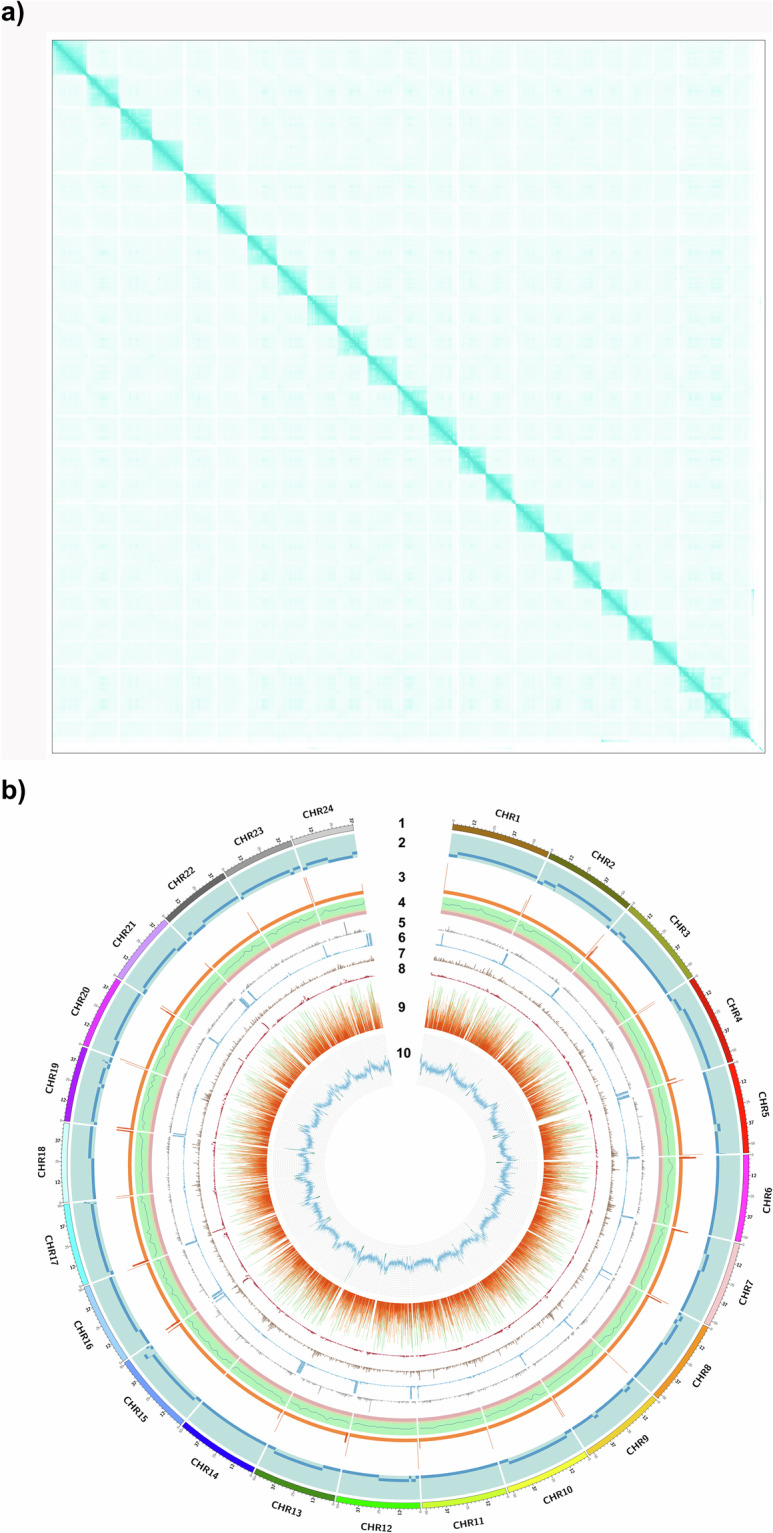
(**a)** Hi-C map representing the 24 Chromosomes of *Etroplus suratensis* genome assembly **(b)** Circos plot representation of the 24 chromosomes of *Etroplus suratensis*. From the outermost: ***Track1***: The 24 chromosomes of the pearlspot genome. ***Track2***: Contigs corresponding to the chromosomes represented as tiles. ***Track3***: Representation of telomeric repeats in log scale at chromosome ends. ***Track4***: Quality values (QV) across the chromosomes shown as line plot with red, yellow and green backgrounds representing ranges 0 - 20, 20 – 30 and 30 -100 respectively. ***Track5 - Track8***: Major repeat elements, SINEs, LINEs, LTRs and DNA Transposons, respectively, shown as scatter plot with a 2 kb sliding window. ***Track9***: Predicted protein-coding genes in chromosomes depicted as highlights with incremental gene lengths of 20 Kb [viz. <20 kb (Very dark orange), 20 kb - 40 kb (dark orange), 40 kb - 60 kb (orange), 60 kb - 80 kb (Light orange) and >80 kb (Green)]. ***Track10***: GC content of pearlspot genome shown as line diagram plotted with 50 kb sliding window. The GC values below 35 and above 45 are shown in dark red color, and remaining in orange color.

**Fig. 3:**
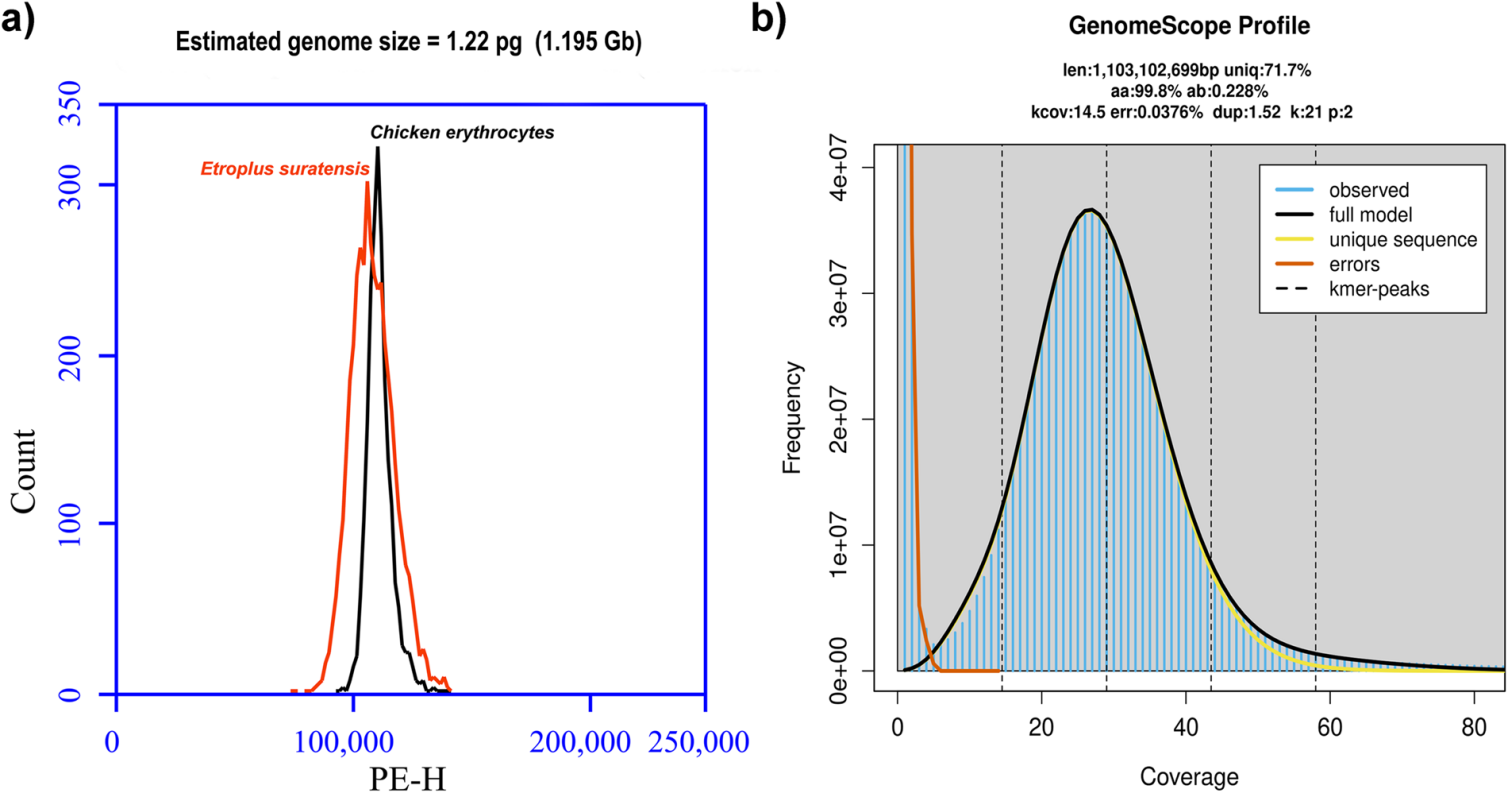
Genome size estimation profiles of *Etroplus suratensis*. (**a)** Flow cytometry principle. Histogram depicting the count of events for *Etroplus suratensis* blood cells and chicken erythrocytes. (**b)** Genome length assessment of *Etroplus suratensis* by k-mer frequency generated using Jellyfish and Genomescope.

In this study, we have used PacBio HiFi technology to generate a highly contiguous genome assembly contigs for pearlspot fish with assembly length of 1.276 Gb and N50 length of 36.16 Mb. Then, the Arima HiC technology was used to order and orient contigs to 24 chromosome-scale scaffolds (Fig. 2**)**. The combination of these two sequencing technologies resulted in the generation of a chromosome-scale genome assembly which is of 1.247 Gb length in 117 scaffolds with N50 length of 51.57 Mb (Table 1**)**. The assembly length is closer to the estimate obtained with flow cytometry method (1.195 Gb) than the estimate obtained with k-mer based analysis (1.103 Gb) using short DNA reads (Fig. 3). A k-mer based analyses estimated the consensus quality value (QV) of 60 for the genome assembly and 98% of assembly length was anchored to 24 chromosomes with telomere ends. Additionally, the haplotype-resolved assemblies generated using a combination of HiFi and HiC reads are of 1.242 Gb and 1.225 Gb with N50 statistic of 51.89 Mb and 51.40 Mb, respectively.

**Table 1:** Genome assembly statistics.

	Primary assembly		Haplotype 1		Haplotype 2	
	Contigs	Scaffolds	Contigs	Scaffolds	Contigs	Scaffolds
**Number**	375	117	340	97	241	64
**Total Length, bp**	1,276,705,672	1,247,975,007	1,271,304,487	1,242,507,214	1,256,813,629	1,225,243,247
**Longest sequence, bp**	60,994,161	60,810,261	54,713,188	59,209,679	52,660,415	60,279,530
**N50, bp**	36,169,634	51,572,587	38,337,071	51,892,000	29,711,116	51,406,431
**L50**	14	12	14	12	16	12
**GC, %**	41.41	41.37	41.4	41.37	41.38	41.35
**Number of N’s per 100 Kbp**	0	0.5	0	0.5	0	0.6

A custom repeat library consisting of 2,112 repeat families obtained through *de novo* modelling of repeat elements in the assembly was used to identify and classify the repeat elements in the pearlspot genome. The repeat elements accounted for 52.96% (Table 2) of the genome predominated by LINEs (20.2%), DNA transposons (16.71%) and LTR elements (3.85%). A strategy that combines evidence generated using Illumina RNAseq reads, PacBio Iso-Seq reads, *ab initio* methods and predicted proteins in related-species genomes resulted in the prediction of 27,192 protein-encoding genes (PEGs) in pearlspot genome. (Table 3). Further, 18,089 non-coding RNAs were detected, with abundant presence of tRNA, ribosomal RNA, spliceosomal RNA, microRNA and Small nucleolar RNA (Supplementary Table 1). The high-quality genome resource would help in specific understanding of salinity tolerance and parental care of pearlspot fish and also the evolution of cichlid fish in general.

**Table 2:** Repeat Profile of *Etroplus suratensis*.

Sequences	117		
Total length	1,247,975,007 bp (1,247,968,707 bp excluding N’s)		
GC level	41.37%		
Bases masked	660,949,585 bp (52.96%)		
**Repeat type**	**Number of elements**	**Length occupied, bp**	**Percntage of sequence**
**Retroelements**	863,047	307,209,769	**24.62**
SINEs:	66,768	7,033,158	0.56
Penelope:	15	2,980	0.00
LINEs:	642,725	252,079,698	20.2
L2/CR1/Rex	250,469	85,118,899	6.82
R1/LOA/Jockey	5,256	1,457,829	0.12
R2/R4/NeSL	12,014	7,702,057	0.62
RTE/Bov-B	297,248	115,031,153	9.22
L1/CIN4	60,645	36,848,449	2.95
LTR elements:	153,554	48,096,913	3.85
BEL/Pao	3,679	2,667,482	0.21
Ty1/Copia	3,056	697,730	0.06
Gypsy/DIRS1	18,419	12,803,482	1.03
Retroviral	18,768	7,736,533	0.62
**DNA transposons**	772,966	208,593,499	16.71
hobo-Activator	84,509	16,079,984	1.29
Tc1-IS630-Pogo	520,926	160,016,019	12.82
MULE-MuDR	377	100,697	0.01
PiggyBac	7,233	1,295,180	0.1
Tourist/Harbinger	28,500	6,478,339	0.52
Other (Mirage, P-element, Transib)	57,376	7,021,251	0.56
**Rolling-circles**	**2,424**	**456,152**	**0.04**
**Unclassified:**	853,729	126,305,226	**10.12**
**Total interspersed repeats:**		**642,111,474**	**51.45**
**Small RNA:**	16,876	2,250,550	**0.18**
**Simple repeats:**	335,317	14,680,430	1.18
**Low complexity:**	33,278	1,699,062	0.14

**Table 3:** Properties of protein-encoding genes.

Property	Statistic
Total sequence length, bp	1,247,975,007
Number of genes	27,192
Number of mRNAs	27,192
Number of exons	235,921
Number of introns	208,729
Number of CDS	27,192
CDS: complete	20,356
CDS: start, no stop	2,603
CDS: stop, no start	2,581
CDS: no stop, no start	1,652
Total gene length, bp	441,547,174
Total mRNA length, bp	441,547,174
Total exon length, bp	39,432,957
Total intron length, bp	402,531,675
Total CDS length, bp	39,432,957
Shortest gene, bp	150
Shortest CDS, bp	138
Longest gene, bp	99,334
Longest CDS, bp	68,233
mean gene length, bp	16,238
mean mRNA length, bp	16,238
mean exon length, bp	167
mean intron length, bp	1,928
mean CDS length, bp	1,450
% of genome covered by genes	35.4
% of genome covered by CDS	3.2
mean mRNAs per gene	1
mean exons per mRNA	9
mean introns per mRNA	8

## Methods

### Specimen for generating sequence data

A single specimen of male pearlspot fish was used to generate the sequence data required for building the genome assembly. The lineage of the specimen was confirmed based on the analysis of the barcoding gene, Cytochrome C Oxidase I (CO I). Briefly, sequence of partial CO I gene of the specimen was generated (MG923355) following amplification with universal primers^6^. The CO I sequence of other accessions under subfamily *Etroplinae* were sourced from BOLD system v4 database^7^ along with *Oreochromis niloticus* accession as outgroup (Supplementary Table 2). The sequences aligned with MUSCLE module of MEGA X^8,9^ were used to build a Maximum Likelihood tree with HKY + G + I model and 1000 bootstrap iterations in MEGA X^9^. (Supplementary Figure S1).

### DNA sequence reads

In this study, three types of DNA sequence data, short reads, long high-fidelity (HiFi) reads and chromatin linked reads (HiC) were generated. The short reads were used to assess the genome properties, the HiFi reads were used for generating genome assembly contigs and HiC reads were used for building assembly scaffolds. Briefly, high molecular weight genomic DNA was isolated from muscle tissue of a single male fish using QIAGEN Genomic-tip 100/G Midi kit (Qiagen, Hilden, Germany). DNA quantity was measured with Qubit 3.0 fluorometer (Thermofisher Scientific, Massachusetts, USA) using DNA HS assay kit (Thermofisher Scientific, Massachusetts, USA) and DNA purity was checked with NanoDrop 2000 (Thermofisher Scientific, Massachusetts, USA). DNA integrity was evaluated on 1% agarose gel and on Femto pulse system (Agilent Technologies, California, USA). DNA shearing was performed on Megaruptor 3 system (Diagenode, Belgium). Three separate sequencing libraries were constructed using the SMRTbell Express template Preparation Kit 2.0 (Pacific Biosciences, California, USA). The libraries were purified using AMPure PB beads (Pacific Biosciences, California, USA) and the purified libraries were treated with SMRTbell Enzyme cleanup kit 2.0 to remove any unbound adapters and damaged DNA. The libraries were size selected using BluePippin (Sage Science, USA) with 0.75% DF Marker S1 High pass Cassette. The size selected libraries were subjected to primer annealing and polymerase binding using Sequel II binding kit 2.2. About 75 to 80 pM of each library was loaded onto individual 8 M SMRT cells (n = 3) and sequenced on PacBio Sequel II system in CCS/HiFi mode to generate polymerase read sequences. Later, the raw polymerase reads were processed with *ccs* algorithm v6.4.0 (–min-passes = 3;–min-snr = 2.5;–min-rq = 0.99) to generate HiFi reads. The HiFi read recovery from polymerase read bases was 50.7% and 4.87% for HiFi read number and HiFi read bases, respectively **(**Table 4**)**.

**Table 4:** Statistics of HiFi reads generated on Pacbio Sequel II using 3 SMRT cells.

	SMRT Cell_1	SMRT Cell_2	SMRT Cell_3
Library loading concentration, pM	80	75	75
Polymerase reads, number	6,534,214	6,496,390	6,642,544
Polymerase reads, bases	669,650,062,929	676,305,302,476	668,450,042,235
Polymerase reads, mean length, bases	102,483	104,104	100,631
Polymerase reads, N50 length, bases	205,598	207,756	201,552
subreads, mean length, bases	8,124	8,253	8,108
subreads, N50 length, bases	10,524	10,616	10,611
HiFi reads, number	3,305,025	3,368,638	3,301,510
HiFi reads, bases	32,386,581,475	33,603,244,498	32,190,388,181
HiFi reads, N50 length, bases	12,098	12,175	12,147
Longest HiFi read length, bases	48,619	47,364	47,060

The same DNA was used to construct a sequencing library with KAPA HyperPlus kit (Basel, Switzerland) as per manufacturers’ protocol. The quality of the library was assessed using Agilent 2100 bioanalyzer (Agilent Technologies, California, USA). The libraries with average insert size of 571 bp were sequenced on Illumina Novaseq6000. These short DNA reads were only used to understand the properties of the genome (Table 5).

**Table 5:** DNA short read data statistics.

	No. of reads	No. of bases	Average length	Q20(%)	Q30(%)
Forward reads	496,268,379	74,936,525,229	151	96.62	92.43
Reverse reads	496,268,379	74,936,525,229	151	96.82	92.27

The HiC library was constructed using Proximo HiC Kit, animal (Phase genomics, USA) as per the manufacturer’s instructions. About 10 nM of library was sequenced using S4 flow cell on Illumina Novaseq6000 in paired-end mode to generate 150 bp linked reads. The restrictions enzymes used to prepare HiC library from fish sample were DpnII, DdeI, HinfI, and MseI (Table 6).

**Table 6:** HiC raw data statistics.

Property	Before filtering		After filtering	
	Read 1	Read 2	Read 1	Read 2
**Total reads**	2,199,442,926	2,199,442,926	1,602,539,154	1,602,539,154
**Total bases**	329,916,438,900	329,916,438,900	238,141,235,712	235,854,087,650
**Q20 bases**	318118560810 (96.424%)	309267991959 (93.7413%)	234884054699 (98.6322%)	230,686,758,252 (97.8091%)
**Q30 bases**	301,565,356,771 (91.4066%)	286148335877 (86.7336%)	226869560126 (95.2668%)	219,0013,31,134 (92.8546%)

In total, 9.975 million HiFi reads (98.18 Gb, 78.7 X), 992.5 million short reads (149.87 Gb, 120.2 X, 92.3% Q30 bases) and 4.398 billion HiC reads (659.83 Gb, 529.1 X, 89% Q30 bases) of DNA sequence data has been generated.

### RNA sequence reads

The RNA sequence reads were generated using specimens of various development stages (1-, 3-, and 15-day old larvae) and tissues (muscle, skin, kidney, liver, stomach, intestine, gill, brain, spleen, testis, and heart) collected from the same adult male Pearlspot fish. Briefly, the total RNA was isolated by using Trizol (DSS Takara, CA, USA) and purified with Nucleospin RNA cleanup kit (Macherey-Nagel, Germany). RNA quantification was performed with Qubit3.0 fluorometer using RNA HS assay kit (ThermoFisher Scientific, Massachusetts, USA) and on Nanodrop 2000. The quality and integrity of RNA was checked on Agilent 2100 bioanalyzer. The cDNA library was prepared with KAPA HyperPrep kit (Roche, Basel, Switzerland) and sequenced on Illumina Novaseq6000 to generate 2 × 150 bp paired-end reads. The raw reads were trimmed with Trimmomatic v0.39^10^ to obtain clean reads with Q30 bases above 90% **(**Table 7**)**.

**Table 7:** Statistics of RNAseq data generated for various adult tissues and various developmental stages of Pearlspot.

	Raw reads	High Quality trimmed reads	GC, %	Q20 bases (%)	Q30 bases (%)
1-day old_R1	24761444	17280870	45	98.49	94.86
1-day old_R2			46	98.08	93.58
3-day old_R1	21824242	12391165	45	97.91	93.19
3-day old_R2			46	97.13	91.30
15-day old_R1	29695710	23911643	46	98.77	95.62
15-day old_R2			46	98.02	93.47
Brain_R1	32745685	24074072	45	98.63	95.22
Brain_R2			46	98.25	94.08
Gill_R1	61234858	50751003	46	98.60	95.15
Gill_R2			46	98.10	93.65
Heart_R1	33201059	26040075	46	99.08	96.52
Heart_R2			46	98.68	95.19
Intestine_R1	39436651	32054953	45	99.17	96.78
Intestine_R2			45	98.31	94.13
Kidney_R1	28133159	23312987	45	98.89	95.95
Kidney_R2			45	98.26	94.07
Liver_R1	32557218	27505400	46	98.98	96.18
Liver_R2			47	97.98	93.39
Muscle_R1	34984634	27002774	48	98.95	96.14
Muscle_R2			48	98.38	94.44
Skin_R1	114178834	79385091	46	98.82	95.71
Skin_R2			46	97.60	92.36
Spleen_R1	31557086	24967590	45	98.75	95.59
Spleen_R2			46	98.33	94.25
Stomach_R1	48763450	38489717	47	98.79	95.68
Stomach_R2			47	97.58	92.47
Testis_R1	40277459	29969267	46	99.11	96.61
Testis_R2			46	98.52	94.72

### Genome size assessment

An assessment of genome size was made on flow cytometry principle with the blood sample following propidium iodide staining in BD Accuri^TM^ C6 flow cytometer^11,12^. The Chicken erythrocytes from BD^TM^ DNA QC Particles kit (BD Biosciences, California, USA) was used as control. The histogram data analyzed with BD Accuri^TM^ C6 Plus software v1.0.23.1 indicated the estimated genome size as 1.22 pg (1.195 Gb) for pearlspot fish (Fig. 3a). The assessment of genome size was also made with DNA sequence reads on k-mer principle. The DNA short reads were subjected to quality trimming with Trimmomatic v0.39^10^ to obtain 78.6 Gb (79X) of clean reads with 96.2% Q30 bases. An assessment of genome properties was made with these clean reads using jellyfish v2.3.0^13^ and GenomeScope v2.0^14^ based on k-mer count and coverage principle. The 21-mer based histogram indicated that the estimated genome length, repeat content and heterozygosity of Pearlspot genome is 1.103 Gb, 28.3% and 0.228%, respectively **(**Fig. 3b**)**.

### Genome assembly

The HiFi reads were initially screened with NCBI foreign contamination screen^15^ to discard contaminants originating from adaptor/vector and foreign organisms. About 78 X coverage of HiFi reads were used to generate assembly contigs with Hifiasm v0.16.1 tool^16^. There were 375 contigs with a total length of 1.276 Gb and N50 length of 36.16 Mb. Then the haplotigs and the overlaps in the primary assembly were removed with purge-dups^17^. Thereafter, about 3.2 billion HiC reads (473.99 Gb, 380 X, 94% Q30 bases) obtained after quality trimming of raw reads with fastp v0.12.4^18^ were used for ordering and orienting the assembly contigs to final scaffolds with YaHS v1.1^19^. The final assembly consisted of 117 scaffolds with a total length of 1.247 Gb and N50 length of 51.57 Mb. The assembly was assessed for its completeness by benchmarking with single-copy orthologs of actinopterygii_odb10 (2021-02-19) using BUSCO v5.7.0^20^. Of the 3,640 BUSCO orthologs, 3,584 were complete and single-copy genes, 29 were complete and duplicated genes, 21 were fragmented genes and 6 were missing genes which indicated that the genome assembly has 99.9% completeness with 0.1% missing genomic regions. The phased assemblies obtained by following the similar methodology were assessed to be 99.2% and 98.7% complete based on BUSCO scores.

### Repeat prediction

The RepeatModeler v 2.0.5 (http://repeatmasker.org/RepeatModeler/) enabling LTR structural analysis was used with *rmblast* v2.14.1 search engine to model and find *de novo* repeat elements in the pearlspot genome. The analysis identified 1,924 RepeatScout/RECON families and 281 LTRPipeline families. After removing redundant LTR families, a custom repeat library with 2,112 repeat families was established. Then, RepeatMasker v 4.1.5^21^ with *rmblast* v2.14.1 search engine was used with custom repeat library to identify and classify the repeat elements in pearlspot genome assembly. The repeat elements accounted for 52.96% **(**Table 2**)** of the genome predominated by LINEs (20.2%), DNA transposons (16.71%) and LTR elements (3.85%).

### Genome annotation

A strategy described earlier^22,23^ that combines evidence generated using Illumina RNAseq reads (generated in this study), PacBio Iso-Seq reads (GenBank accessions, SRR28827909-916), *ab initio* methods and predicted proteins from related-species genomes (Supplementary Table 3) has been used to predict protein-encoding genes (PEGs). Overall, five different evidences were used which were, (1) *ab initio* predictions obtained with AUGUSTUS v3.4.0^24^; (2) predictions with AUGUSTUS v3.4.0^24^ based on hints generated with Iso-Seq reads using GMAP v2017.11.15-4^25^; (3) predictions obtained based on predicted proteins from genomes of related species using BRAKER v2.0.4^26^ and GenomeThreader 1.7.3^27^; (4) Iso-Seq reads derived transcript evidence obtained with GMAP v2017.11.15-4^25^; and (5) RNAseq reads derived transcript evidence obtained with Hisat v2.2.1-4^28^, Stringtie v2.2.1^29^ and TransDecoder v5.7.0. All the five evidences were combined using EVidenceModeler v2.0.0^30^ to arrive at the consensus prediction of PEGs. The Pearlspot genome assembly was predicted to contain 27,192 PEGs with mean exon number of 9 per gene (Table 3). Annotation and pathway analysis^31,32^ of PEGs (Table 8 and Supplementary Figures S2 to S5) were performed by combining results from blastx tool against Actinopterygii (txid7898) dataset of non-redundant database from NCBI, and InterProScan^33^ and EggNOG^34^ mapper module of OmicsBox Tool v3.0.25^35^.

**Table 8:** Annotation statistics of predicted protein-encoding genes.

Attributes	No. of Transcripts	Percentage
**Predicted Genes**	27,192	100.00%
**With Blast Hits**	24,164	88.86%
**With GO Annotation**	21,580	79.36%
**With GO Mapping**	9,923	38.51%
**With Enzyme code**	6,979	29.75%

The identification of noncoding genes in the Pearlspot genome involved aligning the repeat-masked assembly with the Rfam database [http://rfam.xfam.org/], using cmscan from infernal v1.1.2^36^. A total of 18,089 non-coding RNAs were detected, with abundant presence of tRNA, ribosomal RNA, spliceosomal RNA, microRNA and Small nucleolar RNA (Supplementary Table 1).

## Data Records

The raw datasets were deposited under Sequence Read Archive (SRA) at NCBI with the accession numbers, SRR27970333, SRR27999027-029, SRR28233220, SRR28003587-595, SRR28003597-599, SRR28003601-602. The genome was submitted under Genome category at NCBI with the Genome assembly accession number, GCA_041004005.1^37^. All the raw datasets were linked to the Bioproject, PRJNA1076662^38^ and SRA study SRP489803^39^. The genome annotations were submitted to the Figshare repository with the 10.6084/m9.figshare.26303968.v3^32^.

## Technical Validation

**Fig. 4:**
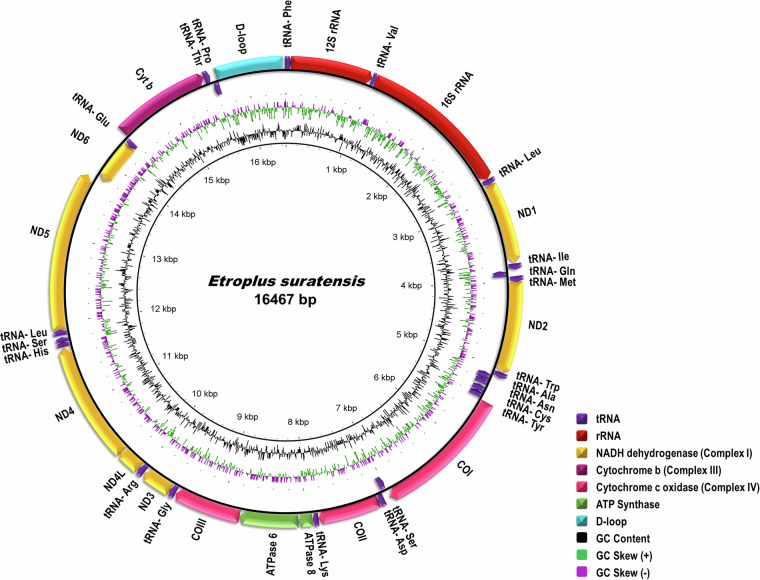
*Etroplus suratensis* mitochondrial genome map depicting the protein coding genes, D-loop, rRNA genes, tRNA genes, GC content and GC Skew.

**Fig. 5:**
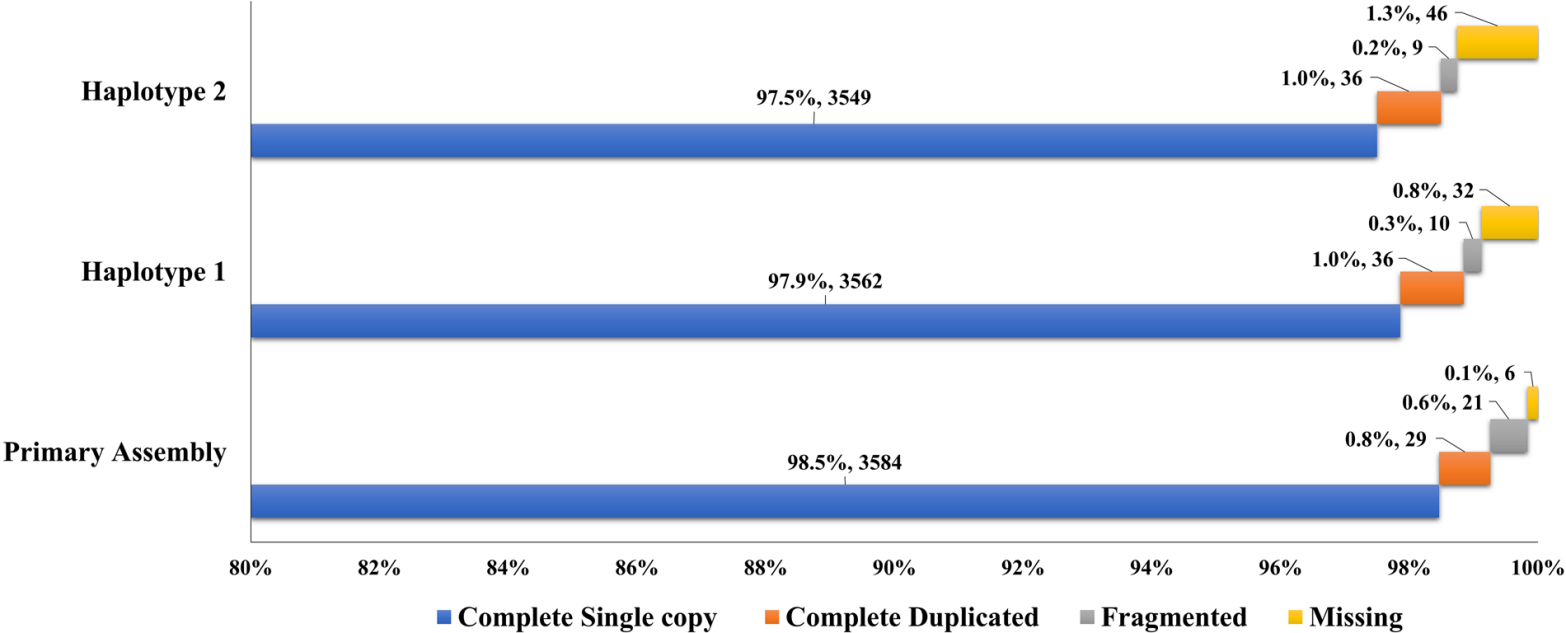
Completeness analysis using BUSCO for *Etroplus suratensis* genome against Actinopterygii_odb10 orthologous database.

**Fig. 6:**
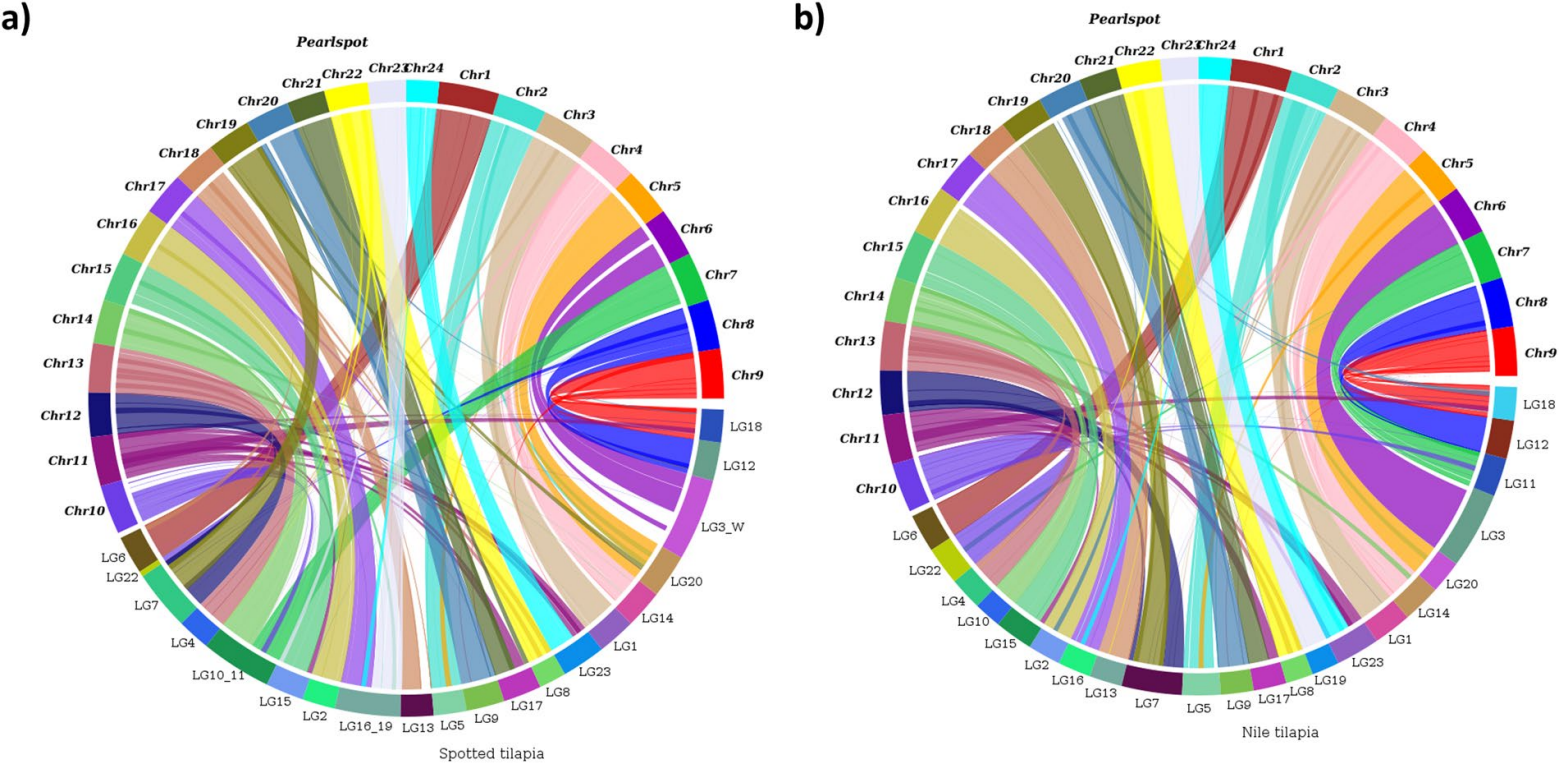
(**a)** Synteny map between Pearlspot and Spotted tilapia (**b)** Synteny map between Pearlspot and Nile tilapia.

The full-length mitochondrial DNA genome (Fig. 4) of 16,467 bp was obtained as a single scaffold in the final assembly suggesting the sufficiency of 78 X coverage of HiFi reads and 380 X coverage of HiC reads. The assembly generated for pearlspot fish is highly contiguous as indicated by contig N50 of 36.16 Mb. The assembly was assessed to be containing 99.3% of complete and 0.6% of the fragmented genes when benchmarked with actinopterygii_odb10 (2021-02-19) lineage using BUSCO v5.7.0^20^ **(**Fig. 5**)**. About 98.03% of the assembly length is represented in the longest 24 scaffolds indicating chromosome-scale nature of the assembly. The consensus quality value and error rate of the genome assembly were assessed to be 60.0762 and 9.82596e-07, respectively when validated with k-mer (31-mer) based procedure executed in Merqury v1.3^40^ indicating high base accuracy of the assembly. The good alignment statistics (Table 9) obtained by aligning RNAseq reads and DNA short reads on to the genome further validated the accuracy of the assembly. The chromosome-scale scaffolds were searched for the presence of telemore repeat sequences using tidk v0.2.0^41^ with Cypriniformes clade (AACCCT). All the scaffolds were observed to be having telomere ends **(**Fig. 2b**,Track 3)**. The genome assembly has shown good synteny **(**Fig. 6**)** with other closely related cichlid fish genomes. About 21,580 (79.36%) of the protein-encoding genes could be annotated functionally (Table 8).

**Table 9:** Read alignment statistics of various short reads generated in the study against the assembled genome of *E. suratensis*.

Data	Accession	Alignment rate (%)
DNA - Illumina short reads	SRR27970333	99.92
RNA - 1-day old	SRR28003598	94.28
RNA - 3-day old	SRR28003597	94.70
RNA - 15-day old	SRR28003595	93.98
RNA - Brain	SRR28003602	91.69
RNA - Gill	SRR28003601	94.87
RNA - Heart	SRR28003594	85.76
RNA - Intestine	SRR28003593	96.01
RNA - Kidney	SRR28003592	94.60
RNA - Liver	SRR28003591	95.84
RNA - Muscle	SRR28003590	96.77
RNA - Skin	SRR28003589	91.42
RNA - Spleen	SRR28003588	96.58
RNA - Stomach	SRR28003587	97.09
RNA - Testis	SRR28003599	96.32

## Supplementary information

Supplementary File 1

## Data Availability

All data processing programs were executed with default parameters unless otherwise specified in the Methods section. There were no custom scripts or code utilized in this study.
