# Supplementary material for: Genome assembly at chromosome scale with telomere ends for Pearlspot, Etroplus suratensis
Source: Sci Data. 2024 Nov 13;11:1226. doi: 10.1038/s41597-024-04096-0 (PMC11560961; doi:10.1038/s41597-024-04096-0)
Supplement: Supplementary file 1 [file 41597_2024_4096_MOESM1_ESM.docx]

**Genome assembly at chromosome scale with telomere ends for Pearlspot, *Etroplus suratensis***

Vinaya Kumar Katneni^1,^*, Karthic Krishnan^1^, Sudheesh K Prabhudas^1^, Roja Jayaraman^1^, Nida Quraishi^1^, Kumaraguru Vasagam^2^, Ashok Kumar Jangam^1^, Jesudhas Raymond Jani Angel^3^, Nimisha Kaikkolante^1^, Kumaravel Jayaraman^2^, Mudagandur S Shekhar^4^

^1^ Centre for Bioinformatics, Nutrition Genetics and Biotechnology Division, ICAR - Central Institute of Brackishwater Aquaculture, No 75, Santhome High Road, MRC Nagar, Chennai, 600 028, Tamil Nadu, India

^2^ Nutrition Genetics and Biotechnology Division, ICAR - Central Institute of Brackishwater Aquaculture, No 75, Santhome High Road, MRC Nagar, Chennai, 600 028, Tamil Nadu, India

^3^ Crustacean Culture Division, ICAR-Central Institute of Brackishwater Aquaculture, No 75, Santhome High Road, MRC Nagar, Chennai, 600028, Tamil Nadu, India

^4^ Aquatic Animal Health and Environment Division, ICAR-Central Institute of Brackishwater Aquaculture, No 75, Santhome High Road, MRC Nagar, Chennai, 600028, Tamil Nadu, India

^*^Corresponding Author: Vinaya Kumar Katneni, email: [Vinaya.Katneni@icar.gov.in](mailto:Vinaya.Katneni@icar.gov.in); [vinayndri@yahoo.com](mailto:vinayndri@yahoo.com)

**Supplementary Table S1**. Summary of non-coding RNAs predicted in the Pearlspot genome

| **Non coding RNA Categories** | | **Count** | |
| --- | --- | --- | --- |
| **ribosomal RNA** | 5.8S ribosomal RNA | 19 | 1918 |
|  | 5S ribosomal RNA | 678 |  |
|  | Archaeal large subunit ribosomal RNA | 6 |  |
|  | Bacterial large subunit ribosomal RNA | 1 |  |
|  | Eukaryotic large subunit ribosomal RNA | 274 |  |
|  | Eukaryotic small subunit ribosomal RNA | 940 |  |
| **microRNA** | microRNA mir-126 | 2 | 96 |
|  | microRNA mir-128 | 2 |  |
|  | microRNA mir-1306 | 1 |  |
|  | microRNA mir-132 | 4 |  |
|  | microRNA mir-137 | 3 |  |
|  | microRNA mir-138 | 3 |  |
|  | microRNA mir-1388 | 1 |  |
|  | microRNA mir-139 | 1 |  |
|  | microRNA mir-140 | 1 |  |
|  | microRNA mir-142 | 4 |  |
|  | microRNA mir-143 | 1 |  |
|  | microRNA mir-144 | 1 |  |
|  | microRNA mir-145 | 1 |  |
|  | microRNA mir-146 | 1 |  |
|  | microRNA mir-153 | 4 |  |
|  | microRNA mir-155 | 1 |  |
|  | microRNA mir-183 | 2 |  |
|  | microRNA mir-184 | 2 |  |
|  | microRNA mir-187 | 1 |  |
|  | microRNA mir-190 | 3 |  |
|  | microRNA mir-193 | 1 |  |
|  | microRNA mir-202 | 1 |  |
|  | microRNA mir-203 | 3 |  |
|  | microRNA mir-204 | 3 |  |
|  | microRNA mir-205 | 2 |  |
|  | microRNA mir-208 | 1 |  |
|  | microRNA mir-21 | 2 |  |
|  | microRNA mir-210 | 1 |  |
|  | microRNA mir-216 | 2 |  |
|  | microRNA mir-217 | 2 |  |
|  | microRNA mir-22 | 3 |  |
|  | microRNA mir-221 | 4 |  |
|  | microRNA mir-223 | 1 |  |
|  | microRNA mir-23 | 4 |  |
|  | microRNA mir-27 | 5 |  |
|  | microRNA mir-301 | 3 |  |
|  | microRNA mir-33 | 2 |  |
|  | microRNA mir-338 | 3 |  |
|  | microRNA mir-363 | 1 |  |
|  | microRNA mir-365 | 1 |  |
|  | microRNA mir-375 | 1 |  |
|  | microRNA mir-454 | 1 |  |
|  | microRNA mir-456 | 1 |  |
|  | microRNA mir-458 | 2 |  |
|  | microRNA mir-460 | 2 |  |
|  | microRNA mir-489 | 1 |  |
|  | microRNA mir-499 | 1 |  |
|  | microRNA mir-551 | 1 |  |
|  | microRNA mir-96 | 2 |  |
| **microRNA precursor family** | let-7 microRNA precursor | 21 | 184 |
|  | mir-101 microRNA precursor family | 2 |  |
|  | mir-103/107 microRNA precursor | 3 |  |
|  | mir-10 microRNA precursor family | 8 |  |
|  | mir-122 microRNA precursor | 1 |  |
|  | mir-124 microRNA precursor family | 6 |  |
|  | mir-1255 microRNA precursor family | 1 |  |
|  | mir-129 microRNA precursor family | 4 |  |
|  | mir-130 microRNA precursor family | 3 |  |
|  | mir-133 microRNA precursor family | 5 |  |
|  | mir-135 microRNA precursor family | 5 |  |
|  | mir-147 microRNA precursor family | 1 |  |
|  | mir-148/mir-152 microRNA precursor family | 2 |  |
|  | mir-15 microRNA precursor family | 4 |  |
|  | mir-16 microRNA precursor family | 3 |  |
|  | mir-1788 microRNA precursor family | 1 |  |
|  | mir-17 microRNA precursor family | 10 |  |
|  | mir-181 microRNA precursor | 4 |  |
|  | mir-182 microRNA precursor family | 2 |  |
|  | mir-192/215 microRNA precursor | 1 |  |
|  | mir-194 microRNA precursor family | 2 |  |
|  | mir-196 microRNA precursor family | 3 |  |
|  | mir-199 microRNA precursor | 4 |  |
|  | mir-19 microRNA precursor family | 5 |  |
|  | mir-1 microRNA precursor family | 3 |  |
|  | mir-214 microRNA precursor family | 3 |  |
|  | mir-2187 microRNA precursor family | 2 |  |
|  | mir-2188 microRNA precursor family | 1 |  |
|  | mir-218 microRNA precursor family | 3 |  |
|  | mir-219 microRNA precursor family | 4 |  |
|  | mir-24 microRNA precursor family | 5 |  |
|  | mir-25 microRNA precursor family | 1 |  |
|  | mir-26 microRNA precursor family | 7 |  |
|  | mir-2985-2 microRNA precursor | 3 |  |
|  | mir-29 microRNA precursor | 7 |  |
|  | mir-30 microRNA precursor | 4 |  |
|  | mir-31 microRNA precursor family | 1 |  |
|  | mir-34 microRNA precursor family | 2 |  |
|  | mir-3618 microRNA precursor family | 1 |  |
|  | miR-430 microRNA precursor family | 1 |  |
|  | mir-455 microRNA precursor family | 2 |  |
|  | mir-459 microRNA precursor family | 1 |  |
|  | MIR6217 microRNA precursor family | 1 |  |
|  | mir-722 microRNA precursor family | 2 |  |
|  | mir-724 microRNA precursor family | 2 |  |
|  | mir-726 microRNA precursor family | 1 |  |
|  | mir-727 microRNA precursor family | 1 |  |
|  | mir-728 microRNA precursor family | 2 |  |
|  | mir-729 microRNA precursor family | 1 |  |
|  | mir-731 microRNA precursor family | 1 |  |
|  | mir-737 microRNA precursor family | 1 |  |
|  | mir-7552 microRNA precursor family | 2 |  |
|  | mir-7 microRNA precursor | 4 |  |
|  | mir-8/mir-141/mir-200 microRNA precursor family | 3 |  |
|  | mir-92 microRNA precursor family | 2 |  |
|  | mir-965 microRNA precursor family | 2 |  |
|  | mir-9/mir-79 microRNA precursor family | 7 |  |
| **Small Cajal body specific RNA** | Small Cajal body specific RNA 1 | 1 | 8 |
|  | Small Cajal body specific RNA 13 | 1 |  |
|  | Small Cajal body specific RNA 14 | 1 |  |
|  | Small Cajal body-specific RNA 2 | 2 |  |
|  | Small Cajal body specific RNA 6 | 2 |  |
|  | Small Cajal body specific RNA 8 | 1 |  |
| **Small nucleolar RNA** | Small nucleolar RNA ACA64 | 2 | 191 |
|  | Small nucleolar RNA MBII-202 | 2 |  |
|  | Small nucleolar RNA R38 | 1 |  |
|  | Small nucleolar RNA SNORA13 | 3 |  |
|  | Small nucleolar RNA SNORA14 | 1 |  |
|  | Small nucleolar RNA SNORA15 | 2 |  |
|  | Small nucleolar RNA SNORA16B/SNORA16A family | 1 |  |
|  | Small nucleolar RNA SNORA17 | 4 |  |
|  | Small nucleolar RNA SNORA18 | 1 |  |
|  | Small nucleolar RNA SNORA19 | 1 |  |
|  | Small nucleolar RNA SNORA22 | 2 |  |
|  | Small nucleolar RNA SNORA23 | 1 |  |
|  | Small nucleolar RNA SNORA26 | 1 |  |
|  | Small nucleolar RNA SNORA29 | 2 |  |
|  | Small nucleolar RNA SNORA2/SNORA34 family | 1 |  |
|  | Small nucleolar RNA SNORA31 | 2 |  |
|  | Small nucleolar RNA SNORA35 | 1 |  |
|  | Small nucleolar RNA SNORA3/SNORA45 family | 3 |  |
|  | Small nucleolar RNA SNORA44 | 1 |  |
|  | Small nucleolar RNA SNORA47 | 1 |  |
|  | Small nucleolar RNA SNORA5 | 3 |  |
|  | Small nucleolar RNA SNORA50 | 1 |  |
|  | Small nucleolar RNA SNORA53 | 2 |  |
|  | Small nucleolar RNA SNORA54 | 1 |  |
|  | Small nucleolar RNA SNORA55 | 1 |  |
|  | Small nucleolar RNA SNORA57 | 2 |  |
|  | Small nucleolar RNA SNORA62/SNORA6 family | 1 |  |
|  | Small nucleolar RNA SNORA63 | 1 |  |
|  | Small nucleolar RNA SNORA65 | 2 |  |
|  | Small nucleolar RNA SNORA68 | 1 |  |
|  | Small nucleolar RNA SNORA69 | 1 |  |
|  | Small nucleolar RNA SNORA70 | 2 |  |
|  | Small nucleolar RNA SNORA71 | 3 |  |
|  | Small nucleolar RNA SNORA73 family | 5 |  |
|  | Small nucleolar RNA SNORA74 | 1 |  |
|  | Small nucleolar RNA SNORA77 | 1 |  |
|  | Small nucleolar RNA SNORA79 | 1 |  |
|  | Small nucleolar RNA SNORA8 | 1 |  |
|  | Small nucleolar RNA SNORA81 | 2 |  |
|  | Small nucleolar RNA SNORA84 | 1 |  |
|  | Small nucleolar RNA SNORA9 | 3 |  |
|  | Small nucleolar RNA SNORD10 | 1 |  |
|  | Small nucleolar RNA SNORD100 | 2 |  |
|  | Small nucleolar RNA SNORD101 | 1 |  |
|  | Small nucleolar RNA SNORD111 | 1 |  |
|  | Small nucleolar RNA SNORD11B | 2 |  |
|  | Small nucleolar RNA SNORD14 | 7 |  |
|  | Small nucleolar RNA SNORD15 | 3 |  |
|  | Small nucleolar RNA SNORD16 | 2 |  |
|  | Small nucleolar RNA SNORD18 | 2 |  |
|  | Small nucleolar RNA SNORD19 | 1 |  |
|  | Small nucleolar RNA SNORD2 | 2 |  |
|  | Small nucleolar RNA SNORD24 | 2 |  |
|  | Small nucleolar RNA SNORD26 | 1 |  |
|  | Small nucleolar RNA SNORD29 | 1 |  |
|  | Small nucleolar RNA SNORD30 | 1 |  |
|  | Small nucleolar RNA SNORD31 | 3 |  |
|  | Small nucleolar RNA SNORD35 | 3 |  |
|  | Small nucleolar RNA SNORD36 | 2 |  |
|  | Small nucleolar RNA SNORD37 | 1 |  |
|  | Small nucleolar RNA SNORD38 | 2 |  |
|  | Small nucleolar RNA SNORD41 | 1 |  |
|  | Small nucleolar RNA SNORD46 | 1 |  |
|  | Small nucleolar RNA SNORD47 | 1 |  |
|  | Small nucleolar RNA SNORD49 | 3 |  |
|  | Small nucleolar RNA SNORD52 | 2 |  |
|  | Small nucleolar RNA SNORD53/SNORD92 | 4 |  |
|  | Small nucleolar RNA SNORD57 | 1 |  |
|  | Small nucleolar RNA SNORD58 | 4 |  |
|  | Small nucleolar RNA SNORD59 | 2 |  |
|  | Small nucleolar RNA SNORD60 | 5 |  |
|  | Small nucleolar RNA SNORD61 | 2 |  |
|  | Small nucleolar RNA SNORD65 | 2 |  |
|  | Small nucleolar RNA SNORD66 | 3 |  |
|  | Small nucleolar RNA SNORD67 | 2 |  |
|  | Small nucleolar RNA SNORD72 | 2 |  |
|  | Small nucleolar RNA SNORD73 | 2 |  |
|  | Small nucleolar RNA SNORD75 | 1 |  |
|  | Small nucleolar RNA SNORD79 | 1 |  |
|  | Small nucleolar RNA SNORD83 | 1 |  |
|  | Small nucleolar RNA SNORD88 | 4 |  |
|  | Small nucleolar RNA SNORD94 | 2 |  |
|  | Small nucleolar RNA SNORD97 | 1 |  |
|  | Small nucleolar RNA SNORD98 | 1 |  |
|  | Small nucleolar RNA SNORD99 | 1 |  |
|  | Small nucleolar RNA snR60/Z15/Z230/Z193/J17 | 2 |  |
|  | Small nucleolar RNA U109 | 1 |  |
|  | Small nucleolar RNA U13 | 1 |  |
|  | Small nucleolar RNA U3 | 10 |  |
|  | Small nucleolar RNA U54 | 1 |  |
|  | Small nucleolar RNA U6-53/MBII-28 | 2 |  |
|  | Small nucleolar RNA U83B | 1 |  |
|  | Small nucleolar RNA U85 | 2 |  |
|  | Small nucleolar RNA Z17 | 1 |  |
|  | Small nucleolar RNA Z195/SNORD33/SNORD32 family | 2 |  |
|  | Small nucleolar SNORD12/SNORD106 | 3 |  |
|  | U7 small nuclear RNA | 3 |  |
|  | U8 small nucleolar RNA | 2 |  |
|  | Z30 small nucleolar RNA | 2 |  |
| **spliceosomal RNA** | U11 spliceosomal RNA | 1 | 467 |
|  | U12 minor spliceosomal RNA | 1 |  |
|  | U1 spliceosomal RNA | 94 |  |
|  | U2 spliceosomal RNA | 101 |  |
|  | U4atac minor spliceosomal RNA | 2 |  |
|  | U4 spliceosomal RNA | 20 |  |
|  | U5 spliceosomal RNA | 56 |  |
|  | U6atac minor spliceosomal RNA | 6 |  |
|  | U6 spliceosomal RNA | 186 |  |
| **Other** | Una+A227:B254L2 LINE 3' element | 33 | 248 |
|  | Vault RNA | 15 |  |
|  | Vertebrate telomerase RNA | 1 |  |
|  | Y RNA | 1 |  |
|  | U1A polyadenylation inhibition element (PIE) | 1 |  |
|  | Nuclear RNase P | 2 |  |
|  | Potassium channel RNA editing signal | 28 |  |
|  | RNase MRP | 1 |  |
|  | Selenocysteine insertion sequence 1 | 4 |  |
|  | Selenocysteine transfer RNA | 1 |  |
|  | Six3os1 conserved region 5 | 1 |  |
|  | Six3os1 conserved region 7 | 1 |  |
|  | 7SK RNA | 13 |  |
|  | Antizyme RNA frameshifting stimulation element | 3 |  |
|  | bZIP non canonical Hac1/Xbp1 intron | 1 |  |
|  | CRISPR RNA direct repeat element | 1 |  |
|  | Editing element of GABA-3 exon 9 | 9 |  |
|  | Microsporidia small subunit ribosomal RNA | 1 |  |
|  | Histone 3' UTR stem-loop | 107 |  |
|  | HOX antisense intergenic RNA myeloid 1 conserved region 2 | 1 |  |
|  | Infectious bronchitis virus D-RNA | 2 |  |
|  | Iron response element I | 2 |  |
|  | Iron response element II | 9 |  |
|  | MALAT1-associated small cytoplasmic RNA/MEN beta RNA | 1 |  |
|  | MAT2A 3'UTR stem loop A | 1 |  |
|  | MAT2A 3'UTR stem loop D | 1 |  |
|  | Metazoan signal recognition particle RNA | 7 |  |
| **tRNA** |  | 14977 | |
| Total | | 18089 | 18089 |

**Supplementary Table S2**: List of the species and their respective accession numbers used for the construction of a Maximum likelihood phylogenetic tree.

| **S.No** | **Species Name** | **Total No. of Accessions used** | **NCBI Accession numbers** |
| --- | --- | --- | --- |
| 1 | *Etroplus suratensis* | 59 | FISBC001, JN228382, KF372997, KF372998, KF372999, KF373000, KF442162, KF442163, KF442164, KF442165, KF442166, KF442167, KF442168, KF442169, KF442170, KF442171, KF442172, KF442173, KF442174, KF442175, KF442176, KF442177, KF442178, KF442179, KF442180, KF442181, KF442182, KF442183, KF442184, KF442185, KF442186, KF442187, KF442188, KF442189, KF442190, KF442191, KF442192, KF442193, KF442194, KF516680, KF516681, KF516682, KU665487, KX371828, MG675620, MG923355, MG923356, MG923357, MG923359, MK336900, MN626365, MW506821, MW595910, MZ312371, NC029832, ON166035, ON166079, ON166100, OP811337* |
| 2 | *Etroplus maculatus* | 6 | MW485078, KC858284, MF601317, MH795977, MZ312370, NC011179 |
| 3 | *Paretroplus damii* | 1 | HQ702350 |
| 4 | *Paretroplus kieneri* | 5 | ON604414, ON604415, ON604416, ON604417, ON604418 |
| 5 | *Paretroplus lamenabe* | 2 | ON604419, ON604420 |
| 6 | *Paretroplus maculatus* | 8 | DSFRE202, MW630758, NC011177, ON604421, ON604422, ON604423, ON604424, ON604425 |
| 7 | *Paretroplus maromandia* | 1 | ON604412 |
| 8 | *Paretroplus menarambo* | 4 | ON604426, ON604427, ON604429, ON604430 |
| 9 | *Paretroplus nourissati* | 5 | DSFRE201, DSMIS112, ON604431, ON604432, ON604433 |
| 10 | *Paretroplus petiti* | 26 | ON604434, ON604435, ON604436, ON604437, ON604438, ON604439, ON604440, ON604441, ON604442, ON604444, ON604445, ON604446, ON604447, ON604448, ON604449, ON604450, ON604451, ON604452, ON604453, ON604454, ON604455, ON604457, ON604458, ON604459, ON604460, ON604461 |
| 11 | *Paretroplus polyactis* | 3 | ON604462, ON604463, ON604464 |
| 12 | *Paretroplus tsimoly* | 1 | ON604413 |
| 13 | *Oreochromis niloticus^#^* | 1 | MK955804^#^ |

* COI sequence generated from the sample used for genome sequencing in the current study

# Outgroup used for construction of phylogenetic tree

**Supplementary Table S3:** List of species, whose protein sequences were used for gene prediction.

| **Assembly Accession** | **Assembly Name** | **Organism Name** | **Annotation Name** | **Assembly Level** | **Scaffold N50** | **Number of Scaffolds** |
| --- | --- | --- | --- | --- | --- | --- |
| GCF_001858045.2 | O_niloticus_UMD_NMBU | *Oreochromis niloticus* | NCBI Oreochromis niloticus Annotation Release 104 | Chromosome | 38839487 | 2459 |
| GCF_000238955.4 | M_zebra_UMD2a | *Maylandia zebra* | NCBI Maylandia zebra Annotation Release 104 | Chromosome | 32660920 | 1689 |
| GCF_013358895.1 | ZZ_aureus | *Oreochromis aureus* | NCBI Oreochromis aureus Annotation Release 101 | Chromosome | 40723988 | 303 |
| GCF_900246225.1 | fAstCal1.2 | *Astatotilapia calliptera* | NCBI Astatotilapia calliptera Annotation Release 100 | Chromosome | 38669361 | 248 |
| GCF_007364275.1 | fArcCen1 | *Archocentrus centrarchus* | NCBI Archocentrus centrarchus Annotation Release 100 | Chromosome | 35590001 | 188 |
| GCF_036321145.2 | Pm_UMD_F_2 | *Pelmatolapia mariae* | GCF_036321145.2-RS_2024_05 | Chromosome | 42111825 | 791 |
| GCF_018398535.1 | NCSU_Asbu1 | *Haplochromis burtoni* | NCBI Haplochromis burtoni Annotation Release 102 | Scaffold | 1410599 | 7420 |
| GCF_000239375.1 | PunNye1.0 | *Pundamilia nyererei* | NCBI Pundamilia nyererei Annotation Release 101 | Scaffold | 2525540 | 7236 |
| GCF_000239395.1 | NeoBri1.0 | *Neolamprologus brichardi* | NCBI Neolamprologus brichardi Annotation Release 101 | Scaffold | 4430025 | 9098 |
| GCF_900408965.1 | fSimDia1.1 | *Simochromis diagramma* | NCBI Simochromis diagramma Annotation Release 100 | Scaffold | 8960300 | 823 |


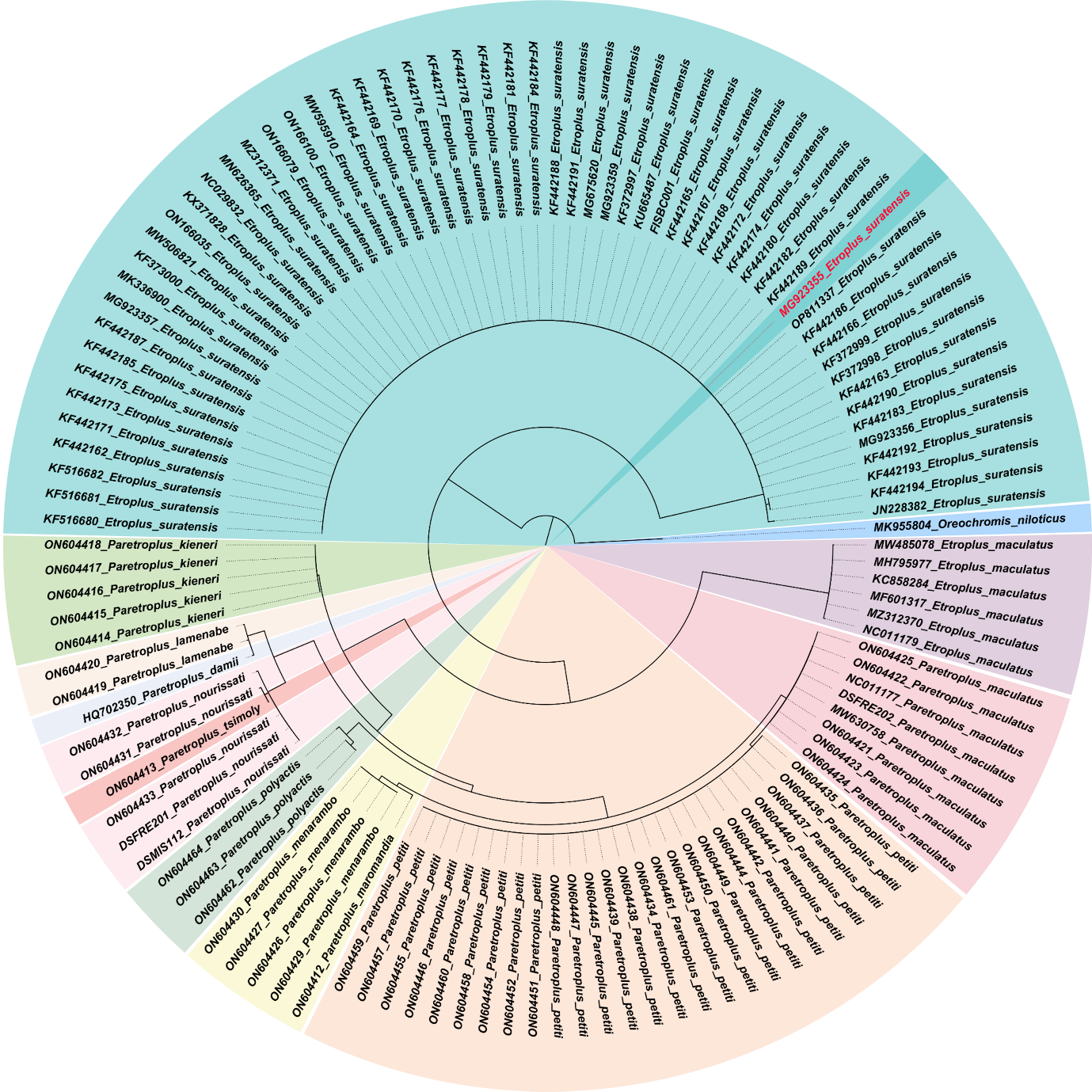


**Supplementary Figure S1**. Maximum likelihood phylogenetic tree based on partial COI gene sequence displaying the specimen used in this study grouping with *Etroplus suratensis* clade.


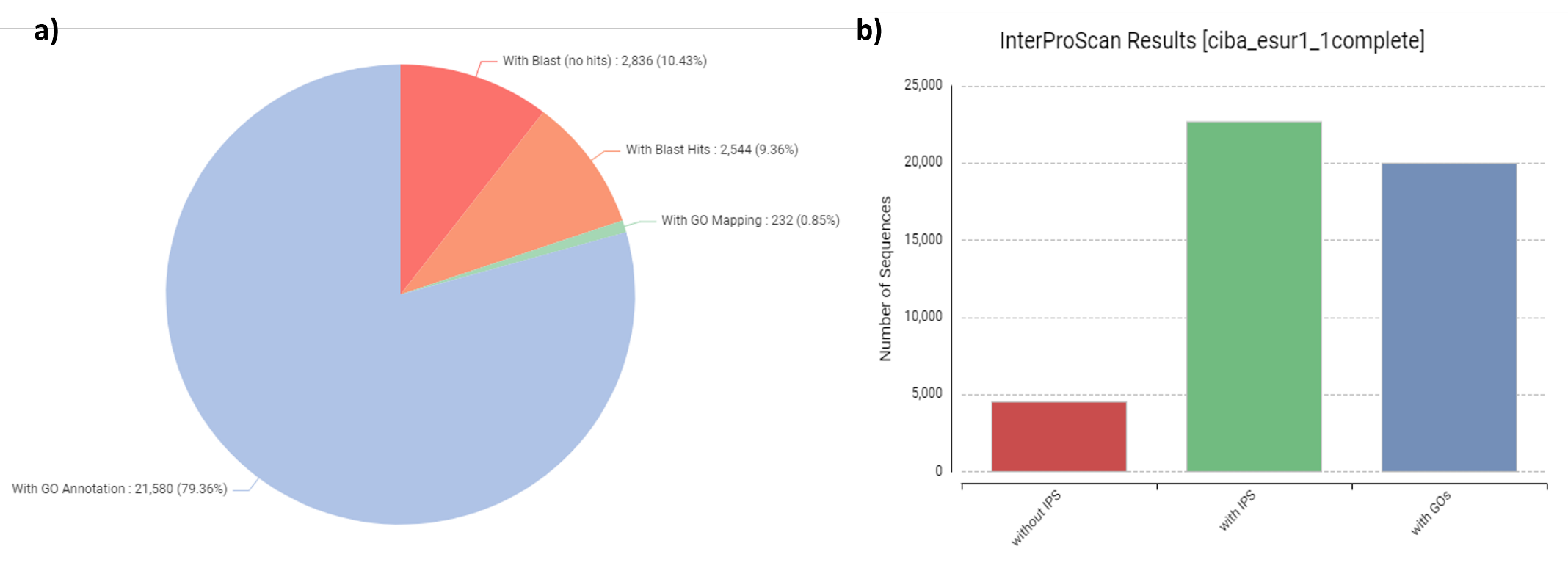


**Supplementary Figure S2:** The distribution of transcripts at various functional annotation levels using Omicsbox tool. **a)** Pie chart depicting the percentage of transcripts with GO annotation, with only GO mapping, with only blast hits and No hits. **b)** Histogram showing the number of transcripts with Interproscan hits, without Interproscan hits and with Gene ontology ID’s.


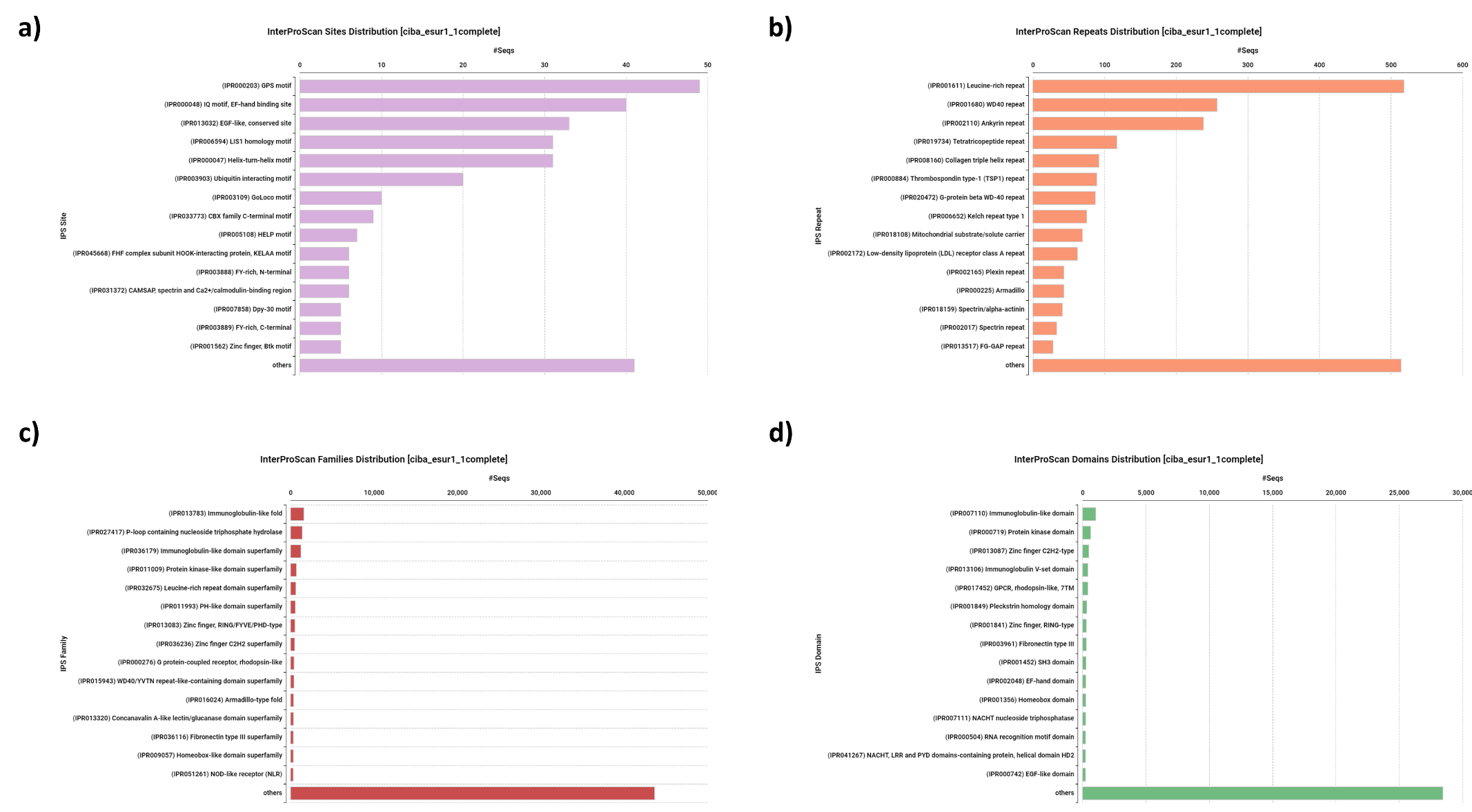


**Supplementary Figure S3** Distribution of transcripts based on different categories of Interproscan results. **a)** Distribution based on sites present on the transcript. **b)** Distribution based on repeats present in the transcripts. **c)** Distribution based on protein families. **d)** Distribution based on domains.


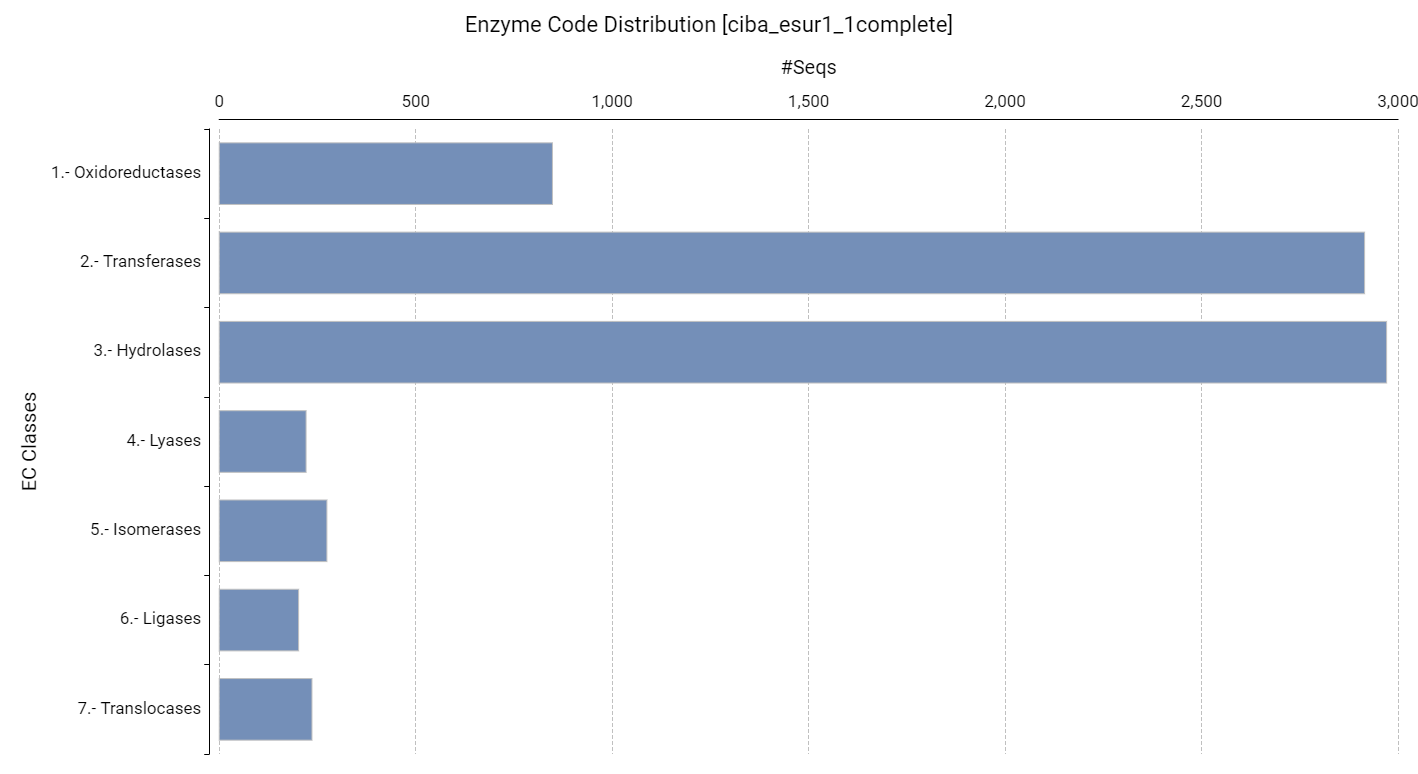


**Supplementary Figure S4:** Histogram depicting the distribution of enzymes identified in the transcripts into different classes


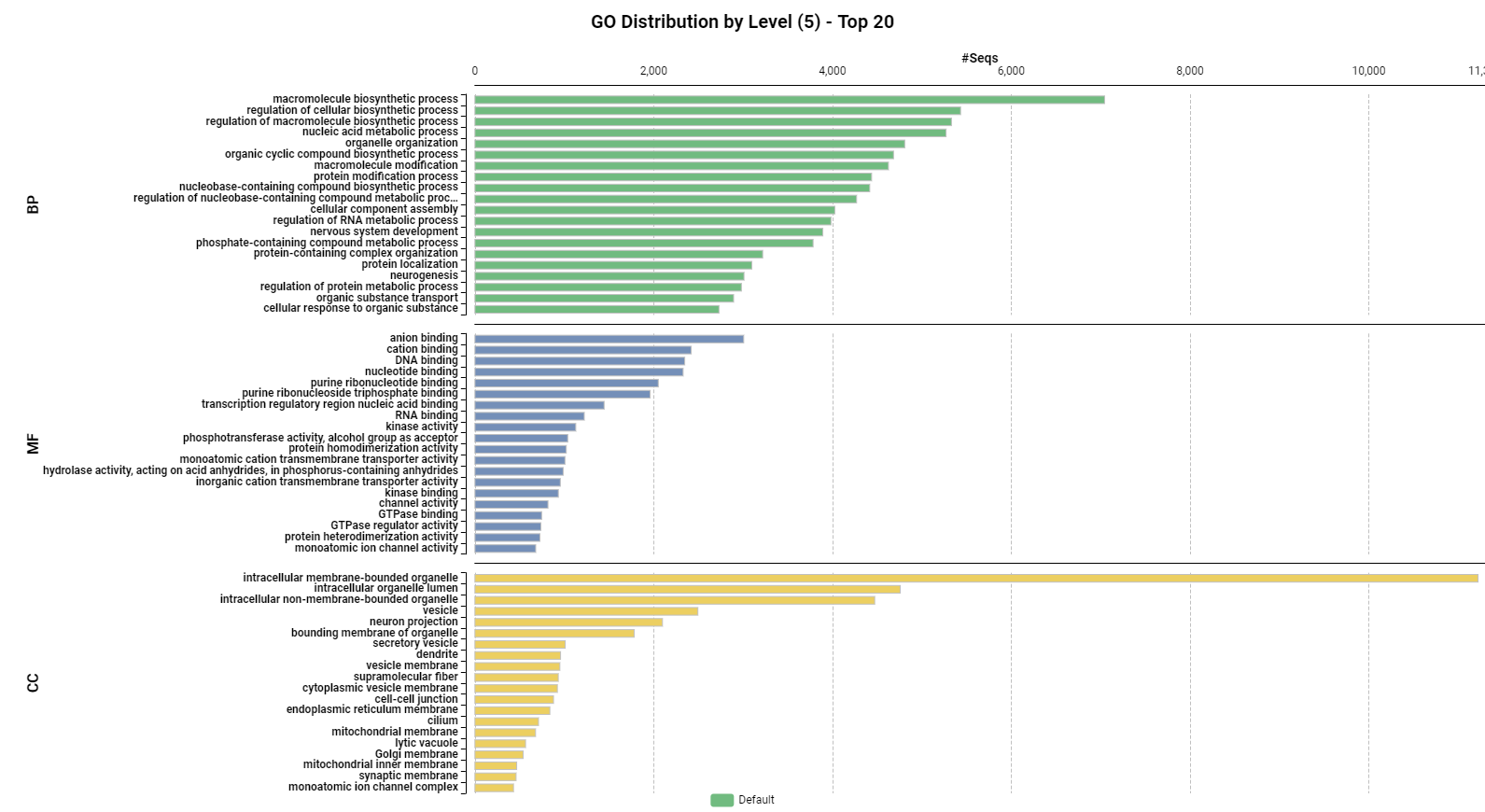


**Supplementary Figure S5:** The Gene Ontology classification of the annotated genes by Level 5 of the predicted genes in *Etroplus suratensis* Genome.
